# Does Hyponatremia Affect the Short-Term Outcomes of Colorectal Cancer Surgery: A Propensity Score Matching Analysis

**DOI:** 10.1155/2022/5109999

**Published:** 2022-09-16

**Authors:** Xiao-Yu Liu, Bin Zhang, Bing Kang, Chao Yuan, Zi-Wei Li, Hua Zhang, Zheng-Qiang Wei, Dong Peng

**Affiliations:** ^1^Department of Gastrointestinal Surgery, The First Affiliated Hospital of Chongqing Medical University, Chongqing, China 400016; ^2^Department of Clinical Nutrition, The First Affiliated Hospital of Chongqing Medical University, Chongqing, China 400016

## Abstract

**Purpose:**

The purpose of the current study is to analyze whether preoperative hyponatremia affected the short-term outcomes of colorectal cancer (CRC) surgery.

**Methods:**

This retrospective study was conducted in a single clinical center where we enrolled patients who underwent primary CRC surgery from January 2011 to December 2021. The short-term outcomes were compared between the hyponatremia group and the normal sodium group using propensity score matching (PSM) analysis.

**Results:**

A total of 6730 cases of patients who received CRC surgery were finally included in this study. There were 184 patients in the hyponatremia group and 6546 patients in the normal sodium group. After 1 : 1 ratio PSM, 184 patients in the normal sodium group were matched to 184 patients in the hyponatremia group. No significant difference was found in baseline information after PSM (*P* > 0.05). After PSM, the hyponatremia group had higher patients with overall complications (*P* = 0.013). Univariate and multivariate logistic regression analysis were conducted to find predictors of complications, and we found that older age (*P* = 0.032, OR = 1.023, 95%CI = 1.002 − 1.044), open surgery (*P* =0.001, OR = 2.300, 95%CI = 1.420 − 3.727), blood loss (*P* = 0.015, OR = 1.002, 95%CI = 1.000 − 1.003), and hyponatremia (*P* = 0.012, OR = 1.856, 95%CI = 1.148 − 3.001) were independent predictors of patients with overall complications.

**Conclusion:**

Hyponatremia was an independent predictor of patients with overall complications after CRC surgery, therefore, the adequate preparation of the patients for surgery remained fundamental.

## 1. Introduction

According to the Global 2020 statistics released by the International Agency for Research on Cancer, colorectal cancer (CRC) has become the third most common cancer and the second leading cause of cancer-related death worldwide [1]. The treatment methods of CRC include surgery, radiotherapy, chemotherapy, immunotherapy, and targeted therapy [2–4]. Surgical resection is still considered to be the first choice for the treatment of CRC. Despite tremendous advances in surgical techniques and medical care strategies, the prognosis of patients with CRC remains to be improved [5–7].

Hyponatremia, which refers to low sodium content in extracellular fluid, is a common electrolyte disturbance in clinical practice [8, 9]. The incidence rate is as high as 15%-30% in acute and chronic hospitalized patients [10, 11]. The clinical manifestations of hyponatremia are mainly related to central nervous system dysfunction, such as headache, nausea, vomiting, lethargy, restlessness, disorientation, and depressive reactions [12]. Complications of severe and rapidly developing hyponatremia include seizures, coma, permanent brain damage, respiratory arrest, brainstem protrusion, and death [13]. Studies have shown that hyponatremia remained an independent predictor of increased mortality risk even mild hyponatremia [14].

Whether preoperative hyponatremia increased the risk of postoperative complications had been studied in orthopedics, cardiac surgery, and head and neck surgery, but there was still a controversy [15–19]. Some studies reported that preoperative hyponatremia was an independent risk factor for complications after cardiac surgery [15], however, other studies reported that it was uncertain whether preoperative correction of hyponatremia was beneficial in reducing complications after total knee arthroplasty [16]. However, it has not been reported whether preoperative hyponatremia affected complications of CRC surgery, therefore, the purpose of the current study was to analyze whether preoperative hyponatremia affected the short-term outcomes of CRC surgery.

## 2. Materials and Methods

### 2.1. Patients

This retrospective study was conducted at a single clinical center where we enrolled patients who underwent primary CRC surgery from January 2011 to December 2021. All relevant procedures were reviewed and approved by the Clinical Research Ethics Committee of the First Affiliated Hospital of Chongqing Medical University (2021-536). This study was conducted in accordance with the Declaration of Helsinki, and all patients signed informed consent.

### 2.2. Inclusion and Exclusion Criteria

A total of 8152 CRC patients who underwent radical CRC surgery were identified in a single teaching hospital. The exclusion criteria were as follows: (1), Patients with recurrent CRC surgery (*n* = 47); (2), Patients who underwent non-R0 CRC surgery (*n* = 22); (3), Incomplete patients' baseline information (*n* = 148); (4), Incomplete Na^+^ examination or hypernatremia (*n* = 1205). Finally, a total of 6730 patients were included.

### 2.3. Surgery Management

All patients with CRC underwent radical resection according to the guidelines of Chinese Society of Clinical Oncology (CSCO) for colorectal cancer, that is total mesorectal excision or complete mesocolic excision, and the postoperative pathology confirmed R0 resection.

### 2.4. Definitions

The value of blood sodium was identified by the first blood test after admission. Normal blood sodium concentration is 135-145 mmol/L, and hyponatremia is defined as blood sodium concentration < 135 mmol/L. Tumor node metastasis (TNM) staging was diagnosed according to the 8^th^ edition of the AJCC [20]. Postoperative complications were defined according to the Clavien-Dindo classification [21], and major complications were defined ≥ III classification complications.

### 2.5. Data Collection

Patients' clinical information, including baseline information and short-term postoperative outcomes, were retrospectively collected from the inpatient system. Baseline information included gender, age, body mass index (BMI), smoking, drinking, underlying diseases (type 2 diabetes mellitus (T2DM), hypertension, and coronary heart disease (CHD)), surgical history, surgical methods, preoperative blood sodium concentration, tumor location, tumor size, and tumor stage. Short-term postoperative outcomes included blood loss, blood transfusion, operative time, postoperative hospital stay, overall complications, and major complications.

### 2.6. Propensity Score Matching (PSM)

To minimize bias in baseline information, PSM was performed between the hyponatremia group and the normal sodium group. Nearest neighbor matching was performed at a 1 : 1 scale without replacement, and a caliper width with 0.01 standard deviation was specified. Baseline information of PSM included gender, age, BMI, smoking, drinking, underlying diseases (T2DM, hypertension, and CHD), surgical history, surgical method, tumor location, tumor size, and tumor stage.

### 2.7. Statistical Analysis

Continuous variables were expressed as mean ± SD, and the independent samples *t*-test was used to compare the differences between the hyponatremia group and the normal sodium group. Frequency variables were represented by *n* (%) and a chi-square test was used. Univariate logistic regression analysis was performed to find potential predictors of complications, and multivariate logistic regression analysis was performed to identify independent predictors of complications (the predictors could be included in the multivariate logistic regression analysis when *P* value was less than 0.05 in univariate logistic regression analysis). Data were analyzed using SPSS (version 22.0) statistical software. A bilateral *P* value of <0.05 was considered statistically significant.

## 3. Results

### 3.1. Patients

**Figure 1:**
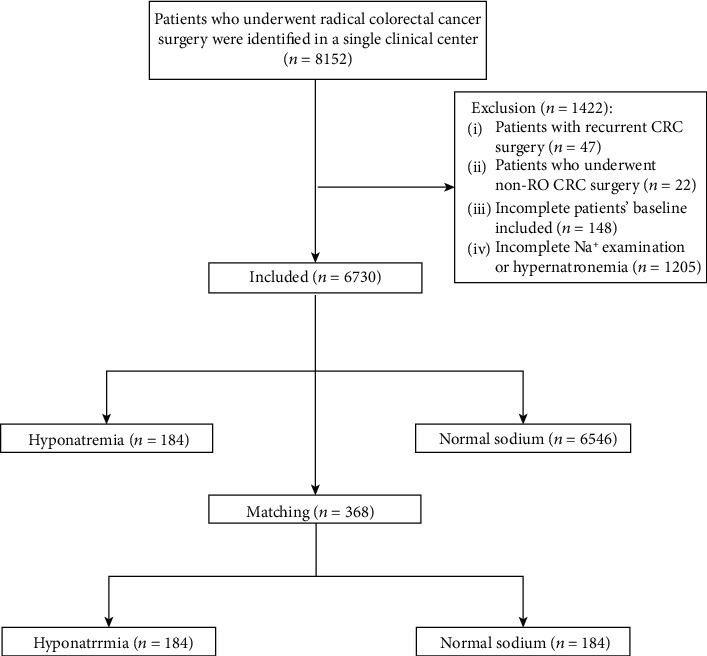
Flow chart of patient selection.

Based on the inclusion and exclusion criteria, 6730 cases of patients who received CRC surgery were finally included in this study. 184 patients were included in the hyponatremia group and 6546 patients were included in the normal sodium group. Given that the classification of the two groups of patients was nonrandom, unbalanced variables might contribute to selection bias, therefore, PSM analysis was used to reduce potential selection bias. After 1 : 1 ratio PSM, 184 patients in the normal sodium group were matched to 184 patients in the hyponatremia group (Figure 1). The baseline information of the included CRC patients was shown in Table 1.

### 3.2. Baseline Information

Comparing the baseline information of the two groups of patients before PSM, the hyponatremia group had an older age (*P* < 0.01), lower BMI (*P* < 0.01), higher proportion of T2DM (*P* < 0.01), higher proportion of open surgery (*P* < 0.01), larger tumors (*P* = 0.001), and higher proportion of colon cancer patients (*P* < 0.01). However, after PSM, there were no significant differences in gender, age, BMI, smoking, drinking, underlying diseases (T2DM, hypertension, and CHD), surgical history, surgical method, tumor location, tumor size, or tumor stage between the two groups (*P* > 0.05) (Table 2).

### 3.3. Short-Term Outcomes

Postoperative short-term outcomes included blood loss, blood transfusion, operative time, postoperative hospital stay, overall complications, and major complications. Before PSM, the hyponatremia group had longer postoperative hospital stay (*P* < 0.01) and higher patients with overall complications (*P* < 0.01). After PSM, the hyponatremia group had higher patients with overall complications (*P* = 0.013) (Table 3).

### 3.4. Univariate and Multivariate Logistic Regression Analysis of Complications

Patients with overall complications in the hyponatremia group were higher than those in the normal sodium group after PSM, therefore, we performed univariate and multivariate logistic regression analysis of matched 368 patients to analyze whether hyponatremia was an independent predictor of patients with overall complications.

Through univariate logistic regression analysis, older age (*P* = 0.007, OR = 1.027, 95%CI = 1.007 − 1.047), open surgery (*P* < 0.01, OR = 2.258, 95%CI = 1.440 − 3.541), T2DM (*P* = 0.033, OR = 1.748, 95%CI = 1.047 − 2.919), CHD (*P* = 0.012, OR = 2.601, 95%CI = 1.238 − 5.466), surgical history (*P* = 0.003, OR = 2.080, 95%CI = 1.279 − 3.384), blood loss (*P* = 0.009, OR = 1.002, 95%CI = 1.000 − 1.003), and hyponatremia (*P* = 0.014, OR = 1.759, 95%CI = 1.123 − 2.753) were predictors of patients with overall complications. In addition, after multivariate logistic regression analysis, we found that older age (*P* = 0.032, OR = 1.023, 95%CI = 1.002 − 1.044), open surgery (*P* = 0.001, OR = 2.300, 95%CI = 1.420 − 3.727), blood loss (*P* = 0.015, OR = 1.002, 95%CI = 1.000 − 1.003), and hyponatremia (*P* = 0.012, OR = 1.856, 95%CI = 1.148 − 3.001) were independent predictors of patients with overall complications (Table 4).

## 4. Discussion

The impact of hyponatremia on the short-term outcomes of surgery was still controversial. Crestanello et al. [15] believed that preoperative hyponatremia was an independent risk factor for postoperative complications after cardiac surgery; Abola et al. [16] reported that it was uncertain whether correction of preoperative hyponatremia symptoms was beneficial in reducing complications after total knee arthroplasty; Hefler-Frischmuth et al. [19] suggested that preoperative hyponatremia could not be an independent prognostic parameter in epithelial ovarian cancer. However, the effect of preoperative hyponatremia on the short-term outcomes after CRC surgery was not reported previously, therefore, the purpose of this study was to investigate the effect of preoperative hyponatremia on short-term outcomes after CRC surgery.

A total of 6730 cases of patients who received CRC surgery were finally included in this study. After 1 : 1 ratio PSM, 184 patients in the normal sodium group were matched to 184 patients in the hyponatremia group, and there were no significant differences in baseline information between the two groups. After multivariate logistic regression analysis, we found that older age, open surgery, blood loss, and hyponatremia were independent predictors of patients with overall complications.

At present, the actual mechanism that preoperative hyponatremia increased postoperative CRC surgery complications was not well understood. We suggested that the underlying pathophysiological mechanisms responsible for these effects might involve hyponatremia-related hypoosmolarity, low extracellular sodium concentration itself, and activation of the neurohormonal axis [15, 22–25]. Na^+^ and their associated anions were the main osmotically active plasma solutes. Decreased blood sodium concentration could directly lead to a decrease in plasma tonicity and induce intracellular transfer of extracellular fluid, thereby altering cell volume and threatening cell viability [15, 22].

Activation of the neurohormonal axis related to sodium concentration mainly included the arginine vasopressin (AVP), the renin-angiotensin-aldosterone system and the sympathetic nervous system. Patients with hyponatremia states were characterized by inappropriately elevated plasma AVP levels [26, 27]. As long as AVP was secreted, it bound to the AVP V2 receptor subtype (V2R) in the collecting duct of the kidney and activated the signal transduction cascade, leading to antidiuresis. If AVP was continuously secreted, it would cause abnormal water retention and persistent hyponatremia [22–28], which would lead to adverse consequences such as heart failure and edema. Due to the potential effects of AVP on V12 and V2 receptors, these receptors could worsen cardiac function by increasing cardiac preload and afterload which led to increased ventricular wall pressure, dilation, and hypertrophy [24]. Loss of intravascular volume (in hypovolemic hyponatremia) and effective intravascular volume (in hypervolemic hyponatremia) activated the neurohumoral axis, leading to increased secretion of AVP, renin, angiotensin II, aldosterone, and catecholamines. While increasing sodium reabsorption, angiotensin II remodeled cardiomyocytes and aldosterone enhanced myocardial fibrosis [22, 29]. In addition, hypovolemic or hypervolemic hyponatremia might affect pathophysiological mechanisms and ultimately lead to adverse outcomes, so it was necessary to actively identify and correct volume problems before surgery.

To our knowledge, there were no previous studies about hyponatremia on the short-term outcomes after CRC surgery. This is the first study to report hyponatremia on the short-term outcomes after CRC surgery, furthermore, PSM is used to minimize the baseline information selection bias.

However, this study still has some limitations. First, this study was a single-center, retrospective study, which might cause selection bias; Second, this study only focused on the short-term outcomes after CRC surgery, and lacked long-term survival analysis; Third, this study only focused on blood sodium concentration, lacked information of the causes of preoperative hyponatremia (such as pseudohyponatremia, syndrome of inappropriate antidiuretic hormone secretion and so on), preoperative correction, postoperative blood sodium concentration, Na^+^ urine, plasma osmolality, and urine osmolality; Fourth, the hyponatremia was associated with a hypovolemic, euvolemic, or hypervolemic picture was not evaluated, and the possible use of drugs capable of causing hyponatremia was not reported because of the limitation of retrospective study (some information was lacking). Therefore, more comprehensive multicenter and prospective randomized controlled studies were needed in the future.

In conclusion, hyponatremia was an independent predictor of overall complications after CRC surgery. The adequate preparation of the patient for surgery remains fundamental, with the achievement of optimal fluid management and volume status; therefore, the identification of the mechanism underlying the hyponatremia and the correct management remain mandatory.

## Figures and Tables

**Table 1:** Clinical characteristics of CRC patients.

Characteristics	No. 6730
Age, (year)	62.8 ± 12.3
Sex	
Male	3971 (59.0%)
Female	2759 (41.0%)
BMI, (kg/m^2^)	22.6 ± 3.2
Smoking	2528 (37.6%)
Drinking	2060 (30.6%)
Hypertension	1681 (25.0%)
T2DM	766 (11.4%)
CHD	312 (4.6%)
Surgery history	1646 (24.5%)
Laparoscopy	5837 (86.7%)
Na^+^	140.8 ± 2.9
Hyponatremia	184 (2.7%)
Tumor location	
Colon	3213 (47.7%)
Rectum	3517 (52.3%)
Tumor size	
<5 cm	4050 (60.2%)
≥5 cm	2680 (39.8%)
TNM stage	
I	1247 (18.5%)
II	2707 (40.2%)
III	2421 (36.0%)
IV	355 (5.3%)
Blood loss, (mL)	106.1 ± 173.2
Blood transfusion	156 (2.3%)
Operation time, (min)	226.3 ± 83.2
Hospital stay, (day)	11.3 ± 8.2
Patients with overall complications	1455 (21.6%)
Patients with major complications	192 (2.9%)

Note: Variables are expressed as the mean ± SD, *n* (%). Abbreviations: T2DM, type 2 diabetes mellitus; BMI, body mass index; CHD, coronary heart disease.

**Table 2:** Baseline characteristics before and after PSM.

Characteristics	Before PSM			After PSM		
	Hyponatremia (184)	Normal sodium (6546)	*P* value	Hyponatremia (184)	Normal sodium (184)	*P* value
Na^+^	131.4 ± 5.0	141.1 ± 2.3	<0.01∗	131.4 ± 5.0	140.6 ± 2.5	<0.01∗
Age (year)	67.6 ± 12.8	62.7 ± 12.3	<0.01∗	67.6 ± 12.8	67.3 ± 12.4	0.810
Sex			0.501			0.831
Male	113 (61.4%)	3858 (58.9%)		113 (61.4%)	111 (60.3%)	
Female	71 (38.6%)	2688 (41.1%)		71 (38.6%)	73 (39.7%)	
BMI (kg/m^2^)	21.5 ± 3.4	22.6 ± 3.2	<0.01∗	21.5 ± 3.4	21.7 ± 3.1	0.522
Smoking	66 (35.9%)	2462 (37.6%)	0.631	66 (35.9%)	60 (32.6%)	0.510
Drinking	50 (27.2%)	2010 (30.7%)	0.305	50 (27.2%)	37 (20.1%)	0.111
Hypertension	51 (27.7%)	1630 (24.9%)	0.384	51 (27.7%)	54 (29.3%)	0.729
T2DM	37 (20.1%)	729 (11.1%)	<0.01∗	37 (20.1%)	44 (23.9%)	0.378
CHD	14 (7.6%)	298 (4.6%)	0.052	14 (7.6%)	17 (9.2%)	0.573
Surgical history	53 (28.8%)	1593 (24.3%)	0.164	53 (28.8%)	42 (22.8%)	0.190
Open surgery	101 (54.9%)	810 (12.4%)	<0.01∗	83 (45.1%)	76 (41.3%)	0.461
Tumor size			0.001∗			0.754
<5 cm	88 (47.8%)	3962 (60.5%)		88 (47.8%)	91 (49.5%)	
≥5 cm	96 (52.2%)	2584 (39.5%)		96 (52.2%)	93 (50.5%)	
Tumor location			<0.01∗			0.551
Colon	134 (72.8%)	3079 (47.0%)		134 (72.8%)	139 (75.5%)	
Rectum	50 (27.2%)	3467 (53.0%)		50 (27.2%)	45 (24.5%)	
Tumor stage			0.202			0.323
I	25 (13.6%)	1222 (18.7%)		25 (13.6%)	15 (8.2%)	
II	77 (41.8%)	2630 (40.2%)		77 (41.8%)	78 (42.4%)	
III	68 (37.0%)	2353 (35.9%)		68 (37.0%)	79 (42.9%)	
IV	14 (7.6%)	341 (5.2%)		14 (7.6%)	12 (6.5%)	

Note: Variables are expressed as the mean ± SD, *n* (%), ^∗^*P* − value < 0.05. Abbreviations: T2DM, type 2 diabetes mellitus; BMI, body mass index; PSM, propensity score matching; CHD, coronary heart disease.

**Table 3:** Short-term outcomes before and after PSM.

Characteristics	Before PSM			After PSM		
	Hyponatremia (184)	Normal sodium (6546)	*P* value	Hyponatremia (184)	Normal sodium (184)	*P* value
Operation time (min)	230.1 ± 82.7	226.2 ± 83.2	0.538	230.1 ± 82.7	224.3 ± 72.4	0.476
Blood loss (mL)	130.3 ± 137.0	105.4 ± 174.1	0.054	130.3 ± 137.0	133.0 ± 187.0	0.877
Hospital stay (day)	13.7 ± 9.0	11.2 ± 8.2	<0.01∗	13.7 ± 9.0	12.9 ± 10.2	0.426
Patients with overall complications	68 (37.0%)	1387 (21.2%)	<0.01∗	68 (37.0%)	46 (25.0%)	0.013∗
Patients with major complications	6 (3.3%)	186 (2.8%)	0.736	6 (3.3%)	6 (3.3%)	1.000

Note: Variables are expressed as the mean ± SD, *n* (%), ^∗^*P* − value < 0.05. Abbreviations: PSM, propensity score matching.

**Table 4:** Univariate and multivariate logistic regression analysis of the patients with overall complications for matched CRC patients.

Risk factors	Univariate logistic regression analysis		Multivariate logistic regression analysis	
	OR (95% CI)	*P* value	OR (95% CI)	*P* value
Age, (year)	1.027 (1.007-1.047)	0.007∗	1.023 (1.002-1.044)	0.032∗
Surgical methods (open/laparoscopic)	2.258 (1.440-3.541)	<0.01∗	2.300 (1.420-3.727)	0.001∗
Sex (male/female)	1.135 (0.723-1.782)	0.581		
BMI, (kg/m^2^)	1.008 (0.941-1.080)	0.820		
Hypertension (yes/no)	1.572 (0.976-2.532)	0.063		
T2DM (yes/no)	1.748 (1.047-2.919)	0.033∗	1.631 (0.935-2.843)	0.085
Surgical history (yes/no)	2.080 (1.279-3.384)	0.003∗	1.636 (0.971-2.755)	0.064
Tumor location (colon/rectum)	1.178 (0.705-1.968)	0.532		
Tumor stage (IV/III/II/I)	0.995 (0.749-1.322)	0.970		
Smoking (yes/no)	0.748 (0.465-1.204)	0.233		
Drinking (yes/no)	1.234 (0.741-2.057)	0.419		
CHD (yes/no)	2.601 (1.238-5.466)	0.012∗	2.282 (1.007-5.170)	0.048
Tumor size (≥ 5/<5), (cm)	1.321 (0.847-2.060)	0.219		
Na^+^ (hyponatremia/normal sodium)	1.759 (1.123-2.753)	0.014∗	1.856 (1.148-3.001)	0.012∗
Blood loss, (mL)	1.002 (1.000-1.003)	0.009∗	1.002 (1.000-1.003)	0.015∗
Operation time, (min)	1.003 (1.000-1.005)	0.077		

Note: ^∗^*P* − value < 0.05, ^∗∗^*P* − value < 0.01. Abbreviations: CRC, colorectal cancer; OR, odds ratio; CI, confidence interval; BMI, body mass index; T2DM, type 2 diabetes mellitus; CHD, coronary heart disease.

## Data Availability

The datasets generated and/or analyzed during the current study are not publicly available but are available from the corresponding author upon reasonable request.

## References

[1] Sung H., Ferlay J., Siegel R. L. (2021). Global cancer statistics 2020: GLOBOCAN estimates of incidence and mortality worldwide for 36 cancers in 185 countries. *CA: a Cancer Journal for Clinicians*.

[2] Liu X. Y., Yuan C., Kang B. (2022). Predictors associated with planned and unplanned admission to intensive care units after colorectal cancer surgery: a retrospective study. *Supportive Care in Cancer*.

[3] Janani B., Vijayakumar M., Priya K. (2022). EGFR-based targeted therapy for colorectal cancer-promises and challenges. *Vaccines*.

[4] Kanani A., Veen T., Søreide K. (2021). Neoadjuvant immunotherapy in primary and metastatic colorectal cancer. *The British Journal of Surgery*.

[5] Dekker E., Tanis P. J., Vleugels J. L. A., Kasi P. M., Wallace M. B. (2019). Colorectal cancer. *Lancet*.

[6] Cheng Y. X., Tao W., Liu X. Y. (2022). Hypertension remission after colorectal cancer surgery: a single-center retrospective study. *Nutrition and Cancer*.

[7] Liu X. Y., Kang B., Cheng Y. X. (2022). The short-term and oncologic outcomes of younger vs older colorectal cancer patients undergoing primary surgery: a propensity score matching analysis. *BMC Cancer*.

[8] Beukhof C. M., Hoorn E. J., Lindemans J., Zietse R. (2007). Novel risk factors for hospital-acquired hyponatraemia: a matched case-control study. *Clinical Endocrinology*.

[9] Adrogué H. J., Madias N. E. (2000). Hyponatremia. *The New England Journal of Medicine*.

[10] DeVita M. V., Gardenswartz M. H., Konecky A., Zabetakis P. M. (1990). Incidence and etiology of hyponatremia in an intensive care unit. *Clinical Nephrology*.

[11] Hawkins R. C. (2003). Age and gender as risk factors for hyponatremia and hypernatremia. *Clinica Chimica Acta*.

[12] Arieff A. I., Guisado R. (1976). Effects on the central nervous system of hypernatremic and hyponatremic states. *Kidney International*.

[13] Ayus J. C., Wheeler J. M., Arieff A. I. (1992). Postoperative hyponatremic encephalopathy in menstruant women. *Annals of Internal Medicine*.

[14] Waikar S. S., Mount D. B., Curhan G. C. (2009). Mortality after hospitalization with mild, moderate, and severe hyponatremia. *The American Journal of Medicine*.

[15] Crestanello J. A., Phillips G., Firstenberg M. S. (2013). Preoperative hyponatremia predicts outcomes after cardiac surgery. *The Journal of Surgical Research*.

[16] Abola M. V., Tanenbaum J. E., Bomberger T. T., Knapik D. M., Fitzgerald S. J., Wera G. D. (2019). Preoperative hyponatremia is associated with reoperation and prolonged length of hospital stay following total knee arthroplasty. *The Journal of Knee Surgery*.

[17] Mc Causland F. R., Wright J., Waikar S. S. (2014). Association of serum sodium with morbidity and mortality in hospitalized patients undergoing major orthopedic surgery. *Journal of Hospital Medicine*.

[18] Carniol E. T., Marchiano E., Brady J. S. (2017). Head and neck microvascular free flap reconstruction: an analysis of unplanned readmissions. *The Laryngoscope*.

[19] Hefler-Frischmuth K., Grimm C., Gensthaler L., Reiser E., Schwameis R., Hefler L. A. (2018). Prognostic value of preoperative hyponatremia and thrombocytosis in patients with epithelial ovarian cancer. *Wiener Klinische Wochenschrift*.

[20] Weiser M. R. (2018). AJCC 8th edition: colorectal cancer. *Annals of Surgical Oncology*.

[21] Clavien P. A., Barkun J., de Oliveira M. L. (2009). The Clavien-Dindo classification of surgical complications. *Annals of Surgery*.

[22] Verbalis J. G., Goldsmith S. R., Greenberg A. (2013). Diagnosis, evaluation, and treatment of hyponatremia: expert panel recommendations. *The American Journal of Medicine*.

[23] Schrier R. W. (2006). Water and sodium retention in edematous disorders: role of vasopressin and aldosterone. *The American Journal of Medicine*.

[24] Barsony J., Sugimura Y., Verbalis J. G. (2011). Osteoclast response to low extracellular sodium and the mechanism of hyponatremia-induced bone loss∗. *The Journal of Biological Chemistry*.

[25] Urbach J., Goldsmith S. R. (2021). Vasopressin antagonism in heart failure: a review of the hemodynamic studies and major clinical trials. *Therapeutic Advances in Cardiovascular Disease*.

[26] Robertson G. L. (2006). Regulation of arginine vasopressin in the syndrome of inappropriate antidiuresis. *The American Journal of Medicine*.

[27] Schrier R. W. (2006). Role of diminished renal function in cardiovascular mortality: marker or pathogenetic factor?. *Journal of the American College of Cardiology*.

[28] Schrier R. W., Berl T. (1975). Nonosmolar factors affecting renal water excretion (first of two parts). *The New England Journal of Medicine*.

[29] Schrier R. W., Abraham W. T. (1999). Hormones and hemodynamics in heart failure. *The New England Journal of Medicine*.

